# The influences of patient's satisfaction with medical service delivery, assessment of medical service, and trust in health delivery system on patient's life satisfaction in China

**DOI:** 10.1186/1477-7525-10-111

**Published:** 2012-09-14

**Authors:** Liyang Tang

**Affiliations:** 1 Department of Economics, School of Economics and Management, Tsinghua University, Beijing, 100084, China

**Keywords:** Life satisfaction, Satisfaction with medical service delivery, Assessment of medical service, Trust in health delivery system, Classification of hospitals, Ordered probit model, China

## Abstract

**Background:**

Patient’s satisfaction with medical service delivery/assessment of medical service/trust in health delivery system may have significant influence on patient’s life satisfaction in China’s health delivery system/in various kinds of hospitals.

The aim of this study was to test whether and to what extent patient’s satisfaction with medical service delivery/patient’s assessments of various major aspects of medical service/various major aspects of patient’s trust in health delivery system influenced patient’s life satisfaction in China’s health delivery system/in various kinds of hospitals.

**Methods:**

This study collaborated with National Bureau of Statistics of China to carry out a 2008 national urban resident household survey in 17 provinces, autonomous regions, and municipalities directly under the central government (N = 3,386), and specified ordered probit models were established to analyze dataset from this household survey.

**Results:**

The key considerations in generating patient’s life satisfaction involved patient’s overall satisfaction with medical service delivery, assessment of doctor-patient communication, assessment of medical cost, assessment of medical treatment process, assessment of medical facility and hospital environment, assessment of waiting time for medical service, trust in prescription, trust in doctor, and trust in recommended medical examination. But the major considerations in generating patient’s life satisfaction were different among low level public hospital, high level public hospital, and private hospital.

**Conclusion:**

The promotion of patient’s overall satisfaction with medical service delivery, the improvement of doctor-patient communication, the reduction of medical cost, the improvement of medical treatment process, the promotion of medical facility and hospital environment, the reduction of waiting time for medical service, the promotion of patient’s trust in prescription, the promotion of patient’s trust in doctor, and the promotion of patient’s trust in recommended medical examination could all help promote patient’s life satisfaction. But their promotion effects were different among low level public hospital, high level public hospital, and private hospital.

## Background

Since the persistent problem “medical service was expensive and difficult to access” in China’s health delivery system greatly reduced patient’s life satisfaction, the Chinese government put a lot of effort into the promotion of patient’s life satisfaction in recent years [1-3]. In China’s health delivery system, patient’s medical experiences in different kinds of hospitals were significantly different, and then effective ways to promote patient’s life satisfaction in different kinds of hospitals were also different, but as researchers have already pointed out, there lacked the holistic and systematic approaches to promote patient’s life satisfaction in various kinds of hospitals [1,2,4].

In fact public hospitals in China can be divided into four categories according to “Governing rules for the management and classification of hospitals” [5]. Level 1 public hospitals are “community hospitals or health clinics that provide direct prevention, treatment, health promotion, and rehabilitation services to participants of a defined community”. Level 2 public hospitals are “area hospitals that provide comprehensive medical and other healthcare services to participants of multiple communities, which may, to a certain degree, also serve as teaching hospitals and research bases”. Level 3 public hospitals are those that “provide high-quality, specialty medical and other healthcare services to participants in a minimum of several areas, and also serve as high-level teaching hospitals and conduct sophisticated research”. Public community health centers serve as complementary health organizations to the three-level public hospital system, their functions are similar to the functions of level 1 public hospitals, but their sizes are smaller and capacities are weaker than those of level 1 public hospitals, and generally speaking, compared with level 1 public hospitals, they mainly provide more junior direct prevention, treatment, health promotion, and rehabilitation services to participants of a defined community.

Compared with public hospitals, a large number of private hospitals were established only after China opened health care market in 2001, and the development of private hospital in China was especially rapid in recent years, in fact the participation of private hospital in health care market alleviated the persistent problem “medical service was expensive and difficult to access” in China’s health delivery system [6].

It was widely accepted that patient generated multi-attribute based responses on her/his life satisfaction in her/his medical experience [7-11]. In fact patient’s life satisfaction in her/his medical experience could be seen as a summarizing response, this summarizing response resulted from patient’s post-treatment cognitive and affective evaluation of medical service delivery, which had significant impact on health-related quality of life given pre-treatment expectation [12-20], and then the directly-related part of the attribute-level responses of patient’s life satisfaction was patient’s satisfaction with medical service delivery. Patient’s assessment of medical service was also one major part of the attribute-level responses of life satisfaction, and from previous studies, major aspects of medical service which patient was most concerned about consisted of the quality of medical treatment process, the quality of doctor-patient communication, the length of waiting time for medical service, the quantity and quality of medical facility, the quality of hospital environment, and medical cost [1,2,21-25]. When patient formed stable trust in health delivery system as response to the unbalanced relationship between patient and doctor/medical institution, patient’s trust in health delivery system influenced her/his attitude towards, cognition on, and response mode with the effectiveness of medical service on improving health-related quality of life to a large extent [26-32], and then another major part of the attribute-level responses of patient’s life satisfaction was composed of several major aspects of patient’s trust in health delivery system, in literature the most important aspects of patient’s trust in health delivery system in China consisted of trust in medical institution, trust in doctor, trust in prescription, and trust in recommended medical examination [1,2,21,23,33]. Other parts of the attribute-level responses of patient’s life satisfaction in her/his medical experience consisted of patient’s certain personal characteristics (involving demographic characteristics, socioeconomic characteristics, medical insurance status, health status, and disease status) [1,2]. Then in order to improve patient’s life satisfaction in China’s health delivery system reform, promoting patient’s satisfaction with medical service delivery, promoting key aspects of medical service which patient was most concerned about, and promoting key aspects of patient’s trust in health delivery system were usually the indirect but effective ways [1,2].

This study was set up to test whether and to what extent patient’s satisfaction with medical service delivery/patient’s assessments of various major aspects of medical service/various major aspects of patient’s trust in health delivery system influenced patient’s life satisfaction in China’s health delivery system/in various kinds of hospitals.

## Methods

### Data

In order to obtain data on patient’s life satisfaction, patient’s overall satisfaction with medical service delivery, patient’s assessment of medical service, and patient’s trust in health delivery system, this study collaborated with National Bureau of Statistics of China to carry out a 2008 national urban resident household survey in 17 provinces, autonomous regions, and municipalities directly under the central government.

This survey adopted the two-stage probability proportional to size (PPS) systematic sampling technique to select a probability sample of 3,508 residents. The face-to-face interviews for this household survey were conducted by professional survey teams from National Bureau of Statistics and local Bureaus of Statistics. The professional investigator usually first invited the resident to fill out the questionnaire on her/his life satisfaction, overall satisfaction with medical service delivery, assessment of medical service, and trust in health delivery system in her/his most recent medical experience. No replacement was made if a selected resident was away, refused to be interviewed, or failed to be interviewed after three attempts. If the resident was unavailable, or disabled in a way that would impede her/him from filling out the questionnaire, another family member who knew the resident best served as the respondent, this family member was also asked to report her/his assessed values of questions in the questionnaire to check bias.

The questionnaire consisted of five sections which had relation to this study. The first section inquired about patient’s certain personal characteristics (involving age, gender, marital status, education, income, employment status, job occupation, health status, medical insurance, reimbursement percentage of medical cost, severity of disease, and stage of disease in her/his most recent medical experience), all these selected personal characteristics were considered as possible influencing factors for patient’s life satisfaction in her/his most recent medical experience. The second section inquired about patient’s life satisfaction in her/his most recent medical experience. The third section inquired about patient’s overall satisfaction with medical service delivery in her/his most recent medical experience. The fourth section inquired about patient’s assessment of medical service in her/his most recent medical experience, the selection of five major aspects of medical service assessment was based on the top five aspects of medical service which patient was most concerned about in the pre-survey for this study (the pre-survey for this study mainly focused on both the selection of major aspects of medical service assessment and the selection of major aspects of patient’s trust in health delivery system) [1,2], specifically speaking, five major aspects of patient’s medical service assessment were composed of assessment of medical treatment process, assessment of doctor-patient communication, assessment of waiting time for medical service, assessment of medical facility and hospital environment, and assessment of medical cost. The fifth section inquired about patient’s trust in health delivery system in her/his most recent medical experience, the selection of four major aspects of patient’s trust in health delivery system was also based on the interview materials in the pre-survey for this study [1,2], specifically speaking, four major aspects of patient’s trust in health delivery system consisted of trust in medical institution, trust in doctor, trust in prescription, and trust in recommended medical examination.

The use of the dataset in this study was approved by National Bureau of Statistics of China.

### Measure of patient’s life satisfaction

The 5-item self-reporting measure that assessed patient’s life satisfaction in her/his most recent medical experience on a scale of 1 to 5 was employed, higher score reflected higher life satisfaction. In this study life satisfaction was operationalized as satisfaction with health-related quality of life and measured with the following question “If you compare your life after the most recent medical experience with your life before the most recent medical experience, is your health-related quality of life after the most recent medical experience better than, equal to or worse than that before the most recent medical experience?”. The option “Much better” was assigned score 5; the option “Somewhat better” was assigned score 4; and the option “Equal” was assigned score 3; the option “Somewhat worse” was assigned score 2; and the option “Much worse” was assigned score 1.

### Measure of patient’s overall satisfaction with medical service delivery

The 5-item self-reporting measure that assessed patient’s overall satisfaction with medical service delivery in her/his most recent medical experience on a scale of 1 to 5 was employed, higher score reflected higher overall satisfaction with medical service delivery: the option “Very satisfied” was assigned score 5; the option “Quite satisfied” was assigned score 4; and the option “Basically satisfied” was assigned score 3; the option “Quite dissatisfied” was assigned score 2; and the option “Very dissatisfied” was assigned score 1.

### Measure of patient’s assessment of medical service

The 5-item self-reporting measures that assessed medical treatment process, doctor-patient communication, waiting time for medical service, medical facility and hospital environment, and medical cost in patient’s most recent medical experience on a scale of 1 to 5 were employed, higher score reflected patient’s higher rating for certain aspect of medical service: the option “Excellent performance in this aspect” was assigned score 5; the option “Good performance in this aspect” was assigned score 4; and the option “General performance in this aspect” was assigned score 3; the option “Bad performance in this aspect” was assigned score 2; and the option “Poor performance in this aspect” was assigned score 1.

### Measure of patient’s trust in health delivery system

The 5-item self-reporting measures that assessed patient’s trust in medical institution, trust in doctor, trust in prescription, and trust in recommended medical examination on a scale of 1 to 5 were employed, higher score reflected higher degree of trust in certain aspect of health delivery system: the option “Have high degree of trust in this aspect” was assigned score 5; the option “Have relatively high degree of trust in this aspect” was assigned score 4; and the option “Have medium degree of trust in this aspect” was assigned score 3; the option “Have relatively low degree of trust in this aspect” was assigned score 2; and the option “Have low degree of trust in this aspect” was assigned score 1.

### Description of ordered probit model

Ordered probit model is especially appropriate for this study, because ordered probit discerns unequal differences between ordinal categories in the dependent variable-patient’s life satisfaction [34-36]. For example, it doesn’t assume that the difference between choosing “Much better” and choosing “Somewhat better” is the same as the difference between choosing “Somewhat worse” and choosing “Much worse”. In fact ordered probit in this study captures the qualitative differences between different degrees of life satisfaction.

In the ordered probit model, the latent evaluation score y_i_ is a linear function of independent variables written as a vector x_i_, here i is sample number, and y_i_ = x_i_*b + ϵ_i_, where b is a vector of coefficients and ϵ_i_ is assumed to follow a standard normal distribution. For an ordered probit model with k cutoff points, define p_j_ (j = 1,2,…,k) as the cutoff points of all y_i_, then y_i_ ≦ p_1_, p_j_ < y_i_ ≦ p_j+1_ (j = 1,2,…,k-1) or y_i_ > p_k_. Following the notation, the ordered probit model is expressed as

(1) $$\text{Prob}\left(y_{i}=y_{0}|x_{i}\right)=Ф\left(p_{1}-x_{i}*b\right)$$

(2) $$\text{Prob}(y_{i}=y_{j}|x_{i})=Ф(p_{j+1}-x_{i}*b)-Ф(p_{j}-x_{i}*b)\;(j=1,2,\ldots ,k-1)$$

(3) $$\text{Prob}\left(y_{i}=y_{k}|x_{i}\right)=1-Ф\left(p_{k}-x_{i}*b\right)$$

where y_j_ (j = 0,1,…,k) is the discrete value of y_i_ and _Ф_ is the cumulative standard normal distribution function [37,38].

The marginal effect of x_i_ can be calculated according to this formula:

(4) $$\partial \text{Prob}\left(y_{i}=y_{0}|x_{i}\right)/\partial x_{i}=-b*\varphi \left(p_{1}-x_{i}*b\right)$$

(5) $$\partial \text{Prob}(y_{i}=y_{j}|x_{i})/\partial x_{i}=-b*(\varphi (p_{j+1}-x_{i}*b)-\varphi (p_{j}-x_{i}*b))\;(j=1,2,\ldots ,k-1)$$

(6) $$\partial \text{Prob}\left(y_{i}=y_{k}|x_{i}\right)/\partial x_{i}=b*\varphi \left(p_{k}-x_{i}*b\right)$$

where φ is the standard normal density function, and based on (4), (5) and (6) the vector of coefficient b can be estimated [37,38].

### Specified ordered probit models

The following three specified ordered probit models were estimated for whole sample/sample in various kinds of hospitals to test whether and to what extent patient’s overall satisfaction with medical service delivery/patient’s assessments of various major aspects of medical service/various major aspects of patient’s trust in health delivery system influenced patient’s life satisfaction in China’s health delivery system/in various kinds of hospitals:

(7) $$\text{Model}\;1:{\text{lifesatisfaction}}_{i}=\beta_{0}+\beta_{1}{\text{overallsatisfaction}}_{i}+\sum_{l}\beta_{l2}z_{\text{li}}+\epsilon_{\text{i}}$$

(8) $$\text{Model}\;2:{\text{lifesatisfaction}}_{i}=\beta_{0}+\sum_{m}\beta_{m1}{\text{assessment}}_{\text{mi}}+\sum_{l}\beta_{l2}z_{\text{li}}+\epsilon_{i}$$

(9) $$\text{Model}\;3:{\text{lifesatisfaction}}_{i}=\beta_{0}+\sum_{n}\beta_{n1}{\text{trust}}_{\text{ni}}+\sum_{l}\beta_{l2}z_{\text{li}}+\epsilon_{i}$$

here i was sample number; lifesatisfaction_i_ was patient’s life satisfaction; overallsatisfaction_i_ was patient’s overall satisfaction with medical service delivery; assessment_mi_ (m = 1,2,…,5) were correspondingly patient’s assessment of medical treatment process, assessment of doctor-patient communication, assessment of waiting time for medical service, assessment of medical facility and hospital environment, and assessment of medical cost; trust_ni_ (n = 1,2,…,4) were correspondingly patient’s trust in medical institution, trust in doctor, trust in prescription, and trust in recommended medical examination; z_li_ were control variables, since patient’s life satisfaction in her/his most recent medical experience may be influenced by certain personal characteristics (involving age, gender, marital status, education, income, employment status, job occupation, health status, medical insurance, reimbursement percentage of medical cost, severity of disease, and stage of disease in her/his most recent medical experience), they were all controlled as dummy variables in the regression models; error term ϵ_i_ was assumed to be distributed normal.

## Results

### Descriptive statistics

In the 2008 national urban resident household survey, a total of 3,386 valid responses were generated, and the response rate was 96.52%. The personal characteristics of the study population were shown in Table 1. The population distribution of each personal characteristic followed the natural distribution of urban resident in China, which was the result of stratified sampling design by National Bureau of Statistics of China.

**Table 1:** Descriptive statistics of personal characteristics

**Dummy variables**	**Descriptions**	**Mean**	**Standard deviation**	**Min**	**Max**
Age dummies	Sample person is between 18–30 years old, 1 = Yes, 0 = Otherwise	0.263	0.441	0	1
	Sample person is between 31–45 years old, 1 = Yes, 0 = Otherwise	0.240	0.427	0	1
	Sample person is between 46–55 years old, 1 = Yes, 0 = Otherwise	0.235	0.424	0	1
Gender dummy	1 = Male, 0 = Female	0.492	0.500	0	1
Marital status dummies	1 = Unmarried, 0 = Otherwise	0.016	0.124	0	1
	1 = Married, 0 = Otherwise	0.909	0.288	0	1
	1 = Divorced, 0 = Otherwise	0.025	0.156	0	1
	1 = Widowed, 0 = Otherwise	0.048	0.213	0	1
Education dummies	1 = Primary School or below, 0 = Otherwise	0.098	0.298	0	1
	1 = Junior high school, 0 = Otherwise	0.259	0.438	0	1
	1 = Senior high school, 0 = Otherwise	0.257	0.437	0	1
	1 = Secondary, 0 = Otherwise	0.103	0.304	0	1
	1 = College, 0 = Otherwise	0.172	0.378	0	1
	1 = University, 0 = Otherwise	0.102	0.303	0	1
Income dummies	1 = Income is less than ¥3390, 0 = Otherwise	0.116	0.321	0	1
	1 = Income is between ¥3390 and ¥5410, 0 = Otherwise	0.118	0.323	0	1
	1 = Income is between ¥5411 and ¥7420, 0 = Otherwise	0.121	0.326	0	1
	1 = Income is between ¥7421 and ¥9374, 0 = Otherwise	0.127	0.333	0	1
	1 = Income is between ¥9375 and ¥11700, 0 = Otherwise	0.127	0.333	0	1
	1 = Income is between ¥11701 and ¥15180, 0 = Otherwise	0.129	0.335	0	1
	1 = Income is between ¥15180 and ¥21860, 0 = Otherwise	0.126	0.332	0	1
Employment status dummies	1 = Employees of state-owned enterprises, 0 = Otherwise	0.321	0.467	0	1
	1 = Employees of various non-state-owned enterprises, 0 = Otherwise	0.193	0.395	0	1
	1 = Urban self-employed and private entrepreneurs, 0 = Otherwise	0.066	0.248	0	1
	1 = Homeworkers, 0 = Otherwise	0.258	0.438	0	1
	1 = Unemployed, to be distributed or other non-employed, 0 = Otherwise	0.022	0.145	0	1
	1 = Students, 0 = Otherwise	0.041	0.198	0	1
	1 = Reemployment of retired or retired personnel, 0 = Otherwise	0.024	0.154	0	1
Job occupation dummies	1 = Professional and technical personnel, 0 = Otherwise	0.029	0.167	0	1
	1 = Managers in government and government related enterprises, 0 = Otherwise	0.141	0.348	0	1
	1 = The clerk and manager, 0 = Otherwise	0.211	0.408	0	1
	1 = Commercial Staff, 0 = Otherwise	0.125	0.330	0	1
	1 = Service staff, 0 = Otherwise	0.004	0.059	0	1
	1 = Farmers, animal husbandry and fishery workers, 0 = Otherwise	0.098	0.297	0	1
	1 = Production workers, transport workers and associated personnel, 0 = Otherwise	0.001	0.030	0	1
Health status dummy	1 = Health status is average or above average, 0 = Otherwise	0.797	0.403	0	1
Medical insurance dummies	1 = Medical insurance for local urban workers, 0 = Otherwise	0.549	0.498	0	1
	1 = Medical insurance for local migrant workers, 0 = Otherwise	0.004	0.062	0	1
	1 = Self-financing medical insurance sponsored by the company or unit, 0 = Otherwise	0.056	0.229	0	1
	1 = Commercial medical insurance bought by employer, 0 = Otherwise	0.006	0.077	0	1
	1 = Privately purchased commercial medical insurance, 0 = Otherwise	0.057	0.232	0	1
	1 = Government funded health care reimbursement, 0 = Otherwise	0.053	0.224	0	1
	1 = The new rural cooperative medical insurance, 0 = Otherwise	0.058	0.233	0	1
	1 = Other medical insurance, 0 = Otherwise	0.074	0.263	0	1
	1 = No medical insurance, 0 = Otherwise	0.137	0.344	0	1
Reimbursement percentage of medical cost dummies	1 = Reimbursement percentage of medical cost is 100%, 0 = Otherwise	0.069	0.253	0	1
	1 = Reimbursement percentage of medical cost is between 70% and 99%, 0 = Otherwise	0.174	0.379	0	1
	1 = Reimbursement percentage of medical cost is between 40% and 69%, 0 = Otherwise	0.148	0.355	0	1
	1 = Reimbursement percentage of medical cost is between 20% and 39%, 0 = Otherwise	0.058	0.234	0	1
	1 = Reimbursement percentage of medical cost is between 1% and 19%, 0 = Otherwise	0.031	0.173	0	1
Severity of disease dummies	1 = Not serious, 0 = Otherwise	0.265	0.441	0	1
	1 = General, 0 = Otherwise	0.517	0.500	0	1
	1 = Serious, 0 = Otherwise	0.172	0.378	0	1
Stage of disease dummies	1 = Emergency and serious disease, 0 = Otherwise	0.176	0.380	0	1
	1 = Non-emergency disease at initial stage, 0 = Otherwise	0.577	0.494	0	1
	1 = Non-emergency disease at medium stage, 0 = Otherwise	0.156	0.363	0	1
	1 = Non-emergency stable disease at late stage, 0 = Otherwise	0.092	0.289	0	1

The descriptive statistics of patient’s life satisfaction, overall satisfaction with medical service delivery, assessment of medical service, and trust in health delivery system in her/his most recent medical experience were presented in Table 2. Averagely speaking, the mean value of patient’s life satisfaction was between the assigned value for the option “Health-related quality of life after the most recent medical experience is somewhat better than that before the most recent medical experience” and the assigned value for the option “Health-related quality of life after the most recent medical experience is equal to that before the most recent medical experience”. The mean value of patient’s overall satisfaction with medical service delivery was between the assigned value for the option “Quite satisfied” and the assigned value for the option “Basically satisfied”. Among patient’s assessments of various major aspects of medical service, the mean value of patient’s assessment of medical treatment process was the highest, and the mean values of patient’s assessment of doctor-patient communication and assessment of medical cost were in the medium level, while the mean values of patient’s assessment of medical facility and hospital environment and assessment of waiting time for medical service were the lowest, but the standard deviation of patient’s assessment of waiting time for medical service was significantly larger than the standard deviations of patient’s assessments of other aspects of medical service. Among various major aspects of patient’s trust in health delivery system, the mean values of patient’s trust in recommended medical examination and trust in doctor were the highest, and the mean value of patient’s trust in prescription was in the medium level, while the mean value of patient’s trust in medical institution was the lowest, but the standard deviation of patient’s trust in medical institution was significantly larger than the standard deviations of other aspects of patient’s trust in health delivery system.

**Table 2:** Descriptive statistics of patient’s life satisfaction, overall satisfaction with medical service delivery, assessment of medical service, and trust in health delivery system

	**Mean**	**Standard deviation**	**Min**	**Max**
Life satisfaction	3.573	0.606	1	5
Overall satisfaction with medical service delivery	3.678	0.774	1	5
Assessment of medical treatment process	3.580	0.750	1	5
Assessment of doctor-patient communication	3.494	0.778	1	5
Assessment of waiting time for medical service	3.139	1.030	1	5
Assessment of medical facility and hospital environment	3.397	0.683	1	5
Assessment of medical cost	3.448	0.804	1	5
Trust in medical institution	2.760	1.349	1	5
Trust in doctor	4.144	0.986	1	5
Trust in prescription	3.482	0.911	1	5
Trust in recommended medical examination	4.189	0.996	1	5

### Regression results for whole sample

Results of specified ordered probit model 1–3 for whole sample were presented in Table 3. From the significance and size of the coefficient for patient’s overall satisfaction with medical service delivery/patient’s assessment of certain major aspect of medical service/certain major aspect of patient’s trust in health delivery system, the influences of patient’s overall satisfaction with medical service delivery/patient’s assessments of various major aspects of medical service/various major aspects of patient’s trust in health delivery system on patient’s life satisfaction in China’s health delivery system were revealed as follows.

**Table 3:** Results of model 1–3 for whole sample

	**(1)**	**(2)**	**(3)**
	**Life satisfaction**		
Overall satisfaction with medical service delivery	2.741***		
	(26.00)		
Assessment of medical treatment process		0.546***	
		(9.17)	
Assessment of doctor-patient communication		0.629***	
		(10.84)	
Assessment of waiting time for medical service		0.260***	
		(5.80)	
Assessment of medical facility and hospital environment		0.513***	
		(14.98)	
Assessment of medical cost		0.621***	
		(11.58)	
Trust in medical institution			0.0156
			(1.06)
Trust in doctor			0.156***
			(7.77)
Trust in prescription			0.157***
			(7.52)
Trust in recommended medical examination			0.147***
			(7.89)
Age dummies	Yes	Yes	Yes
Gender dummy	Yes	Yes	Yes
Marital status dummies	Yes	Yes	Yes
Education dummies	Yes	Yes	Yes
Income dummies	Yes	Yes	Yes
Employment status dummies	Yes	Yes	Yes
Job occupation dummies	Yes	Yes	Yes
Health status dummy	Yes	Yes	Yes
Medical insurance dummies	Yes	Yes	Yes
Reimbursement percentage of medical cost dummies	Yes	Yes	Yes
Severity of disease dummies	Yes	Yes	Yes
Stage of disease dummies	Yes	Yes	Yes
Cutoff point 1	4.086***	5.133***	−0.0979
	(8.78)	(9.89)	(−0.26)
Cutoff point 2	7.885***	7.222***	1.153***
	(15.27)	(13.72)	(3.08)
Cutoff point 3	12.15***	10.62***	3.170***
	(20.46)	(18.88)	(8.39)
Cutoff point 4	15.20***	13.80***	4.819***
	(22.54)	(22.49)	(12.56)
Number of observations	3386	3386	3386
Log pseudo-likelihood	3160.5	1740.7	300.0

t statistics in parentheses, * p < 0.10,** p < 0.05,*** p < 0.01.

Patient’s overall satisfaction with medical service delivery had very significant positive influence on her/his life satisfaction in China’s health delivery system, 1-point increase in patient’s overall satisfaction with medical service delivery would even lead to 2.741-point increase in patient’s life satisfaction. Among patient’s assessments of various major aspects of medical service, patient’s assessment of doctor-patient communication and assessment of medical cost had the largest positive influences on her/his life satisfaction, and the positive influences of patient’s assessment of medical treatment process and assessment of medical facility and hospital environment on her/his life satisfaction were in the medium level, while patient’s assessment of waiting time for medical service had the smallest positive influence on her/his life satisfaction. Among various major aspects of patient’s trust in health delivery system, patient’s trust in prescription, trust in doctor, and trust in recommended medical examination had significant positive influences on her/his life satisfaction, while only patient’s trust in medical institution had no significant influence on her/his life satisfaction.

### Regression results for sample in various kinds of hospitals

Results of specified ordered probit model 1–3 for sample in various kinds of hospitals were correspondingly presented in Table 4, Table 5, and Table 6. From the significance and size of the coefficient for patient’s overall satisfaction with medical service delivery/patient’s assessment of certain major aspect of medical service/certain major aspect of patient’s trust in health delivery system, the influences of patient’s overall satisfaction with medical service delivery/patient’s assessments of various major aspects of medical service/various major aspects of patient’s trust in health delivery system on patient’s life satisfaction in various kinds of hospitals were revealed as follows.

**Table 4:** Results of model 1 for sample in various kinds of hospitals

	**(1)**	**(2)**	**(3)**	**(4)**	**(5)**
	**Group for community health center**	**Group for level 1 hospital**	**Group for level 2 hospital**	**Group for level 3 hospital**	**Group for private hospital**
	**Life satisfaction**				
Overall satisfaction with medical service delivery	2.246***	2.463***	2.860***	3.122***	6.837
	(10.67)	(11.27)	(13.19)	(11.52)	(0.03)
Age dummies	Yes	Yes	Yes	Yes	Yes
Gender dummy	Yes	Yes	Yes	Yes	Yes
Marital status dummies	Yes	Yes	Yes	Yes	Yes
Education dummies	Yes	Yes	Yes	Yes	Yes
Income dummies	Yes	Yes	Yes	Yes	Yes
Employment status dummies	Yes	Yes	Yes	Yes	Yes
Job occupation dummies	Yes	Yes	Yes	Yes	Yes
Health status dummy	Yes	Yes	Yes	Yes	Yes
Medical insurance dummies	Yes	Yes	Yes	Yes	Yes
Reimbursement percentage of medical cost dummies	Yes	Yes	Yes	Yes	Yes
Severity of disease dummies	Yes	Yes	Yes	Yes	Yes
Stage of disease dummies	Yes	Yes	Yes	Yes	Yes
Cutoff point 1	4.464**	4.863**	3.853***	4.640***	13.47
	(2.14)	(2.07)	(4.06)	(5.50)	(0.03)
Cutoff point 2	8.074***	8.554***	7.716***	8.894***	21.37
	(3.79)	(3.62)	(7.29)	(8.50)	(0.03)
Cutoff point 3	11.84***	12.63***	12.09***	13.52***	29.94
	(5.32)	(5.13)	(9.92)	(10.52)	(0.04)
Cutoff point 4	14.37***	15.73***	15.23***	16.82***	37.03
	(6.22)	(6.13)	(11.00)	(11.04)	(0.04)
Number of observations	620	443	936	938	449
Log pseudo-likelihood	328.7	442.8	925.0	946.9	452.8

t statistics in parentheses, * p < 0.10,** p < 0.05,*** p < 0.01.

**Table 5:** Results of model 2 for sample in various kinds of hospitals

	**(1)**	**(2)**	**(3)**	**(4)**	**(5)**
	**Group for community health center**	**Group for level 1 hospital**	**Group for level 2 hospital**	**Group for level 3 hospital**	**Group for private hospital**
	**Life satisfaction**				
Assessment of medical treatment process	0.789***	0.646***	0.461***	0.433***	0.275
	(6.05)	(5.68)	(3.13)	(2.60)	(1.53)
Assessment of doctor-patient communication	0.575***	0.659***	0.676***	0.757***	0.517***
	(5.57)	(4.62)	(5.57)	(4.16)	(2.75)
Assessment of waiting time for medical service	0.175	0.200*	0.257**	0.452***	0.237
	(1.40)	(1.95)	(2.50)	(5.04)	(1.51)
Assessment of medical facility and hospital environment	0.621***	0.550***	0.509***	0.449***	0.527***
	(8.18)	(8.20)	(5.72)	(4.83)	(4.55)
Assessment of medical cost	0.525***	0.633***	0.691***	0.854***	0.533***
	(5.48)	(5.39)	(4.27)	(6.04)	(3.18)
Age dummies	Yes	Yes	Yes	Yes	Yes
Gender dummy	Yes	Yes	Yes	Yes	Yes
Marital status dummies	Yes	Yes	Yes	Yes	Yes
Education dummies	Yes	Yes	Yes	Yes	Yes
Income dummies	Yes	Yes	Yes	Yes	Yes
Employment status dummies	Yes	Yes	Yes	Yes	Yes
Job occupation dummies	Yes	Yes	Yes	Yes	Yes
Health status dummy	Yes	Yes	Yes	Yes	Yes
Medical insurance dummies	Yes	Yes	Yes	Yes	Yes
Reimbursement percentage of medical cost dummies	Yes	Yes	Yes	Yes	Yes
Severity of disease dummies	Yes	Yes	Yes	Yes	Yes
Stage of disease dummies	Yes	Yes	Yes	Yes	Yes
Cutoff point 1	5.085***	4.747***	5.750***	5.896***	2.654
	(3.76)	(3.80)	(4.64)	(6.86)	(1.41)
Cutoff point 2	9.085***	7.223***	7.710***	7.772***	5.445***
	(4.39)	(5.60)	(6.15)	(8.93)	(2.80)
Cutoff point 3	12.56***	10.98***	11.53***	11.02***	8.792***
	(5.89)	(7.77)	(8.62)	(11.66)	(4.39)
Cutoff point 4	15.73***	13.94***	15.33***	14.13***	11.64***
	(7.05)	(8.73)	(10.70)	(13.59)	(5.40)
Number of observations	620	443	936	938	449
Log pseudo-likelihood	269.1	244.6	572.5	479.8	159.1

t statistics in parentheses, * p < 0.10,** p < 0.05,*** p < 0.01.

**Table 6:** Results of model 3 for sample in various kinds of hospitals

	**(1)**	**(2)**	**(3)**	**(4)**	**(5)**
	**Group for community health center**	**Group for level 1 hospital**	**Group for level 2 hospital**	**Group for level 3 hospital**	**Group for private hospital**
	**Life satisfaction**				
Trust in medical institution	0.0435	0.0610	−0.00226	0.0286	0.00469
	(0.86)	(1.47)	(−0.08)	(1.06)	(0.11)
Trust in doctor	0.196***	0.190***	0.179***	0.129***	0.119**
	(3.96)	(4.87)	(2.81)	(3.32)	(2.20)
Trust in prescription	0.176***	0.182***	0.185***	0.204***	0.174***
	(4.51)	(4.82)	(4.58)	(4.55)	(2.83)
Trust in recommended medical examination	0.0989*	0.170***	0.201***	0.254***	0.127*
	(1.74)	(4.34)	(5.28)	(4.30)	(1.82)
Age dummies	Yes	Yes	Yes	Yes	Yes
Gender dummy	Yes	Yes	Yes	Yes	Yes
Marital status dummies	Yes	Yes	Yes	Yes	Yes
Education dummies	Yes	Yes	Yes	Yes	Yes
Income dummies	Yes	Yes	Yes	Yes	Yes
Employment status dummies	Yes	Yes	Yes	Yes	Yes
Job occupation dummies	Yes	Yes	Yes	Yes	Yes
Health status dummy	Yes	Yes	Yes	Yes	Yes
Medical insurance dummies	Yes	Yes	Yes	Yes	Yes
Reimbursement percentage of medical cost dummies	Yes	Yes	Yes	Yes	Yes
Severity of disease dummies	Yes	Yes	Yes	Yes	Yes
Stage of disease dummies	Yes	Yes	Yes	Yes	Yes
Cutoff point 1	0.230	2.615**	−0.900	−0.316	−1.206
	(0.16)	(2.22)	(−1.18)	(−0.54)	(−0.88)
Cutoff point 2	1.987	4.049***	0.288	0.861	0.184
	(1.46)	(3.40)	(0.38)	(1.49)	(0.14)
Cutoff point 3	4.103***	6.285***	2.276***	2.882***	2.234
	(2.99)	(5.21)	(2.98)	(4.93)	(1.64)
Cutoff point 4	5.899***	7.873***	3.889***	4.529***	3.835***
	(4.26)	(6.39)	(5.06)	(7.56)	(2.79)
Number of observations	620	443	936	938	449
Log pseudo-likelihood	50.22	83.18	96.92	92.45	35.82

t statistics in parentheses, * p < 0.10,** p < 0.05,*** p < 0.01.

Patient’s overall satisfaction with medical service delivery had very significant positive influence on her/his life satisfaction in all kinds of public hospitals, and the positive influence of patient’s overall satisfaction with medical service delivery on patient’s life satisfaction in high level public hospital was usually larger than that in low level public hospital, but in private hospital patient’s overall satisfaction with medical service delivery had no significant influence on her/his life satisfaction. Patient’s assessment of medical treatment process, assessment of doctor-patient communication, assessment of medical facility and hospital environment, and assessment of medical cost all had significant positive influences on her/his life satisfaction in all kinds of public hospitals, but only in high level public hospital patient’s assessment of waiting time for medical service had significant positive influence on her/his life satisfaction. The positive influences of patient’s assessment of doctor-patient communication and assessment of medical cost on patient’s life satisfaction in high level public hospital were usually larger than those in low level public hospital, while the positive influences of patient’s assessment of medical treatment process and assessment of medical facility and hospital environment on patient’s life satisfaction in low level public hospital were usually larger than those in high level public hospital. In private hospital only patient’s assessment of doctor-patient communication, assessment of medical facility and hospital environment, and assessment of medical cost had significant positive influences on patient’s life satisfaction. Patient’s trust in doctor, trust in prescription, and trust in recommended medical examination all had significant positive influences on her/his life satisfaction in all kinds of hospitals, while patient’s trust in medical institution had no significant influence on her/his life satisfaction in any kind of hospital. The positive influences of patient’s trust in prescription and trust in recommended medical examination on patient’s life satisfaction in high level public hospital were usually larger than those in low level public hospital, while the positive influence of patient’s trust in doctor on patient’s life satisfaction in low level public hospital was usually larger than that in high level public hospital.

## Discussion

### Main findings of this study

In China’s health delivery system, patient’s overall satisfaction with medical service delivery was the most important consideration in generating her/his life satisfaction in all kinds of public hospitals. Among five major aspects of medical service which patient was most concerned about, patient’s assessment of doctor-patient communication (particularly in high level public hospital and private hospital), assessment of medical cost (particularly in high level public hospital and private hospital), assessment of medical treatment process (particularly in low level public hospital), assessment of medical facility and hospital environment (particularly in low level public hospital and private hospital), and assessment of waiting time for medical service (particularly in high level public hospital) were all key considerations in generating her/his life satisfaction. And among four major aspects of patient’s trust in health delivery system, patient’s trust in prescription (particularly in high level public hospital and private hospital), trust in doctor (particularly in low level public hospital and private hospital), and trust in recommended medical examination (particularly in high level public hospital) were all important considerations in generating her/his life satisfaction.

### What is already known on this topic

Previous studies separately showed that patient’s satisfaction with medical service delivery, patient’s assessment of medical service, and patient’s trust in health delivery system were all important considerations when patient comprehensively evaluated health-related quality of life in her/his medical experience to generate her/his life satisfaction [2,3,21-25,33]. But up till now the influences of patient’s satisfaction with medical service delivery, assessment of medical service, and trust in health delivery system on patient’s life satisfaction haven’t been studied under a systematic and comprehensive framework, and then their relative importance in influencing patient’s life satisfaction hasn’t been made clear, most importantly, differences of their relative importance in influencing patient’s life satisfaction among different kinds of hospitals haven’t been studied before.

In most of these previous studies, patient’s assessment of medical service/patient’s trust in health delivery system was usually taken as a whole, while little study subdivided patient’s assessment of medical service/patient’s trust in health delivery system into different aspects and further studied their different influencing effects on patient’s life satisfaction [2,3,21-25,33].

Many previous studies mentioned above obtained their conclusions only through qualitative analysis and authors’ experiences, while only a small number of studies adopted strict quantitative analysis, and a large number of conclusions in these studies were usually drawn on the basis of a small range of patients, then the robustness and universality of conclusions in previous studies remained controversial.

### What this study adds

The most important contribution of this study was that the influences of patient’s overall satisfaction with medical service delivery/patient’s assessments of various major aspects of medical service/various major aspects of patient’s trust in health delivery system on patient’s life satisfaction in China’s health delivery system/in various kinds of hospitals were studied under a systematic and comprehensive framework for the first time. The second most important contribution of this study was that in order to perform the strict quantitative analysis, this study collaborated with National Bureau of Statistics of China to carry out the 2008 national urban resident household survey which focused on both health care delivery and patient satisfaction for the first time in China. The third most important contribution was that different key influencing factors for patient’s life satisfaction in different kinds of hospitals were found for the first time in China. The fourth most important contribution was that the robustness and universality of findings in this study were much better than those in previous studies.

On the basis of major findings in this study, in China’s future health delivery system reform, the following inspirations on effective ways to promote patient’s life satisfaction in China’s health delivery system/in various kinds of hospitals were found. In all kinds of public hospitals, the promotion of patient’s overall satisfaction with medical service delivery could greatly promote patient’s life satisfaction. The improvement of doctor-patient communication (particularly in high level public hospital and private hospital), the reduction of medical cost (particularly in high level public hospital and private hospital), the improvement of medical treatment process (particularly in low level public hospital), the promotion of medical facility and hospital environment (particularly in low level public hospital and private hospital), and the reduction of waiting time for medical service (particularly in high level public hospital) could all help promote patient’s life satisfaction. The promotion of patient’s trust in prescription (particularly in high level public hospital and private hospital), the promotion of patient’s trust in doctor (particularly in low level public hospital and private hospital), and the promotion of patient’s trust in recommended medical examination (particularly in high level public hospital) would all be beneficial to the promotion of patient’s life satisfaction.

### Limitations of this study

Several limitations should be noted. First, the response rate of the 2008 national urban resident household survey was 96.52%, and then the collected sample slightly deviated from the stratified sampling design, which may have slight influence on the above findings. Second, there may be other potential influencing factors for patient’s life satisfaction that were not contained or controlled in this study, which may affect the influence of patient’s overall satisfaction with medical service delivery/patient’s assessment of medical service/patient’s trust in health delivery system on patient’s life satisfaction. Third, in fact patient’s assessments of various major aspects of medical service/various major aspects of patient’s trust in health delivery system interrelated with each other, which may affect their relative influences on patient’s life satisfaction.

### Target audience

Findings in this study may prove useful for both practitioners in various kinds of hospitals and regulators in various regulatory organizations. Practitioners in different kinds of hospitals could find the different effective ways to promote patient’s life satisfaction through promoting corresponding key influencing factors (among patient’s overall satisfaction with medical service delivery, major aspects of medical service which patient was most concerned about, and major aspects of patient’s trust in health delivery system). Regulators in different regulatory organizations could implement different effective interventions for the promotion of patient’s life satisfaction through targeted laws, policies, regulations, and measures. For example, in order to effectively promote patient’s life satisfaction in public hospital, the improvement of medical treatment process and the promotion of medical facility and hospital environment through targeted laws, policies, regulations, and measures should be emphasized particularly in low level public hospital, while the improvement of doctor-patient communication and the reduction of medical cost through targeted laws, policies, regulations, and measures should be emphasized particularly in high level public hospital.

## Conclusion

In all kinds of public hospitals, the promotion of patient’s overall satisfaction with medical service delivery could greatly promote patient’s life satisfaction. The improvement of doctor-patient communication (particularly in high level public hospital and private hospital), the reduction of medical cost (particularly in high level public hospital and private hospital), the improvement of medical treatment process (particularly in low level public hospital), the promotion of medical facility and hospital environment (particularly in low level public hospital and private hospital), and the reduction of waiting time for medical service (particularly in high level public hospital) could all help promote patient’s life satisfaction. The promotion of patient’s trust in prescription (particularly in high level public hospital and private hospital), the promotion of patient’s trust in doctor (particularly in low level public hospital and private hospital), and the promotion of patient’s trust in recommended medical examination (particularly in high level public hospital) would all be beneficial to the promotion of patient’s life satisfaction.

## Competing interests

The author declares that he has no competing interests.

## Authors’ contributions

LT carried out the data collection, performed the statistical analysis, conceived and drafted the manuscript. LT also approved the final manuscript. All authors read and approved the final manuscript.
